# Structural Analysis of Laminarin-Derived Oligosaccharides Produced by Transglycosylation of Exo-β-1,3-glucanase ScEXG1 from *Saccharomyces cerevisiae*

**DOI:** 10.1021/acs.jafc.5c04471

**Published:** 2025-12-24

**Authors:** Szu-Yu Kuo, Chih-Chieh Lin, Hsin-Jo Chang, Reuben Wang, Pei-Yin Lin, Ting-Jang Lu, Yi-Chen Lo

**Affiliations:** † Institute of Food Science and Technology, 33561 National Taiwan University, No. 1, Section 4, Roosevelt Rd., Taipei 106319, Taiwan; ‡ Institute of Food Safety and Health, College of Public Health, 33561 National Taiwan University, Taipei 100025, Taiwan; § Master of Public Health Program (MPH), College of Public Health, 33561 National Taiwan University, Taipei 100025, Taiwan; ∥ College of Bioresources and Agriculture, Joint Center for Instruments and Researches, No. 81, Changxing St., Da-an Dist., Taipei 106038, Taiwan

**Keywords:** transglycosylation, glycosyl hydrolases (GHs), *Saccharomyces cerevisiae* EXG1 (ScEXG1), laminarin-derived oligosaccharides, structural characterization

## Abstract

Exo-β-1,3-glucanase EXG1 (ScEXG1) from *Saccharomyces cerevisiae* hydrolyzes β-1,3-glucans and exhibits transglycosylation activity, enabling biocatalytic synthesis of prebiotic oligosaccharides. In this study, recombinant ScEXG1–6 × His was tested with laminaribiose as both the donor and acceptor. Under low-enzyme dosage, transglycosylation products were detected across a range of substrate concentrations (4.38–32.85 mM), with 21.9 mM laminaribiose producing 270.70 ± 10.32 nmol/mL of the main trisaccharide product-DP3 (DP3) after 3 h. Structural elucidation by HPLC-ESI-MS/MS identified DP3 as β-Glc-(1→6)-β-Glc-(1→3)-Glc based on glycosidic and cross-ring fragmentation patterns. Two additional tetrasaccharides were also characterized as β-Glc-(1→3)-β-Glc-(1→6)-β-Glc-(1→3)-Glc (DP4–1) and β-Glc-(1→6)-β-Glc-(1→3)-β-Glc-(1→3)-Glc (DP4–2). The findings highlight ScEXG1’s ability to produce structurally diverse β-glucan oligosaccharides under mild conditions, thereby expanding enzymatic strategies for prebiotic oligosaccharide production.

## Graphical abstract

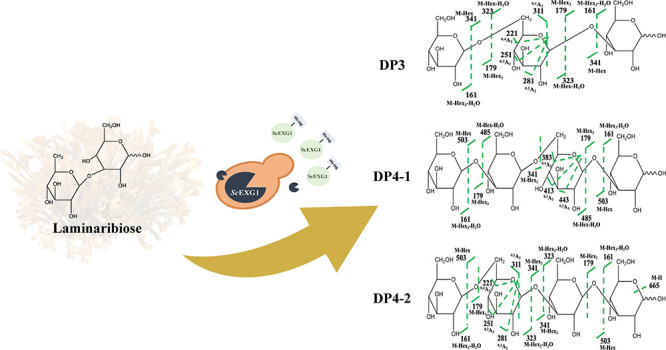

## 1 Introduction

Functional oligosaccharides, which are short-chain carbohydrates resistant to digestion by human gut enzymes, offer a wide range of health benefits. They can serve as prebiotics or food additives to enhance the nutritional value of food products.^1^ Laminarin, an underexplored marine carbohydrate primarily composed of β (1→3)-glucans found in brown algae, and its associated oligosaccharides have gained recognition for their immune modulatory, antitumor, antioxidant, and other biological activities.^2–6^ Recent research has highlighted the immunoreceptor Dectin-1’s heightened binding affinity to β(1→3)-glucan-oligosaccharides with mono-(1→6)-β side-chain branching, triggering innate immune responses against fungal pathogens. This discovery underscores the potential functional role of structure-specific oligosaccharides as prebiotics.^7^

*Saccharomyces cerevisiae* exo-β-1,3-glucanase (ScEXG1), a glucoside hydrolase family 5 (GH5) enzyme,^8^ catalyzes β (1→3)-glucan hydrolysis via a retaining Koshland double-displacement mechanism, releasing β-d-glucose from the nonreducing ends.^8,9^ Some studies have indicated ScEXG1’s ability to hydrolyze β(1→6) glycosidic linkages in oligo-/polysaccharides or glycosyl attached to aglycones such as gentibiose, pustulan,^8^ and mogrosides.^10^ Moreover, glycosyl hydrolases are known to catalyze transglycosylation reactions that compete with hydrolysis, allowing the formation of new glycosidic bonds under controlled conditions or through engineered mutants.^11,12^ Given ScEXG1’s involvement in the cell wall glucan assembly, it has been proposed to also function as a transglycosylase. A previous study demonstrated that ScEXG1 could generate oligosaccharides with a degree of polymerization higher than the initial laminaribiose substrate;^8^ however, the structural identity of these products, the optimal reaction conditions for transglycosylation, and the oligosaccharide yield were not fully characterized. The determination of oligosaccharide structures is pivotal for understanding the structure–function relationship. In addition to nuclear magnetic resonance (NMR),^13,14^ structural analysis and identification of xyloglucan oligosaccharides have been achieved through a combination of high-performance anion-exchange chromatography, reversed-phase high-performance liquid chromatography, or capillary electrophoresis coupled with matrix-assisted laser ionization tandem time-of-flight mass spectrometers or electrospray ionization mass spectrometry (ESI-MS^*n*^).^15^ More recently, Lin et al. demonstrated the diverse profiles of linear and branched galacto-oligosaccharides using porous graphitic carbon liquid chromatography-orbitrap tandem mass spectrometry (PGC-LC-Orbitrap-MS/MS).^16^ This advanced analytical approach enabled the detection of hexose oligosaccharides up to DP12 and facilitated the determination of fragmentation patterns and linkages from the reducing end to the nonreducing end of oligosaccharides through cross-ring cleavage ions.^16^ These advanced analytical tools hold promise for the identification and analysis of oligosaccharides, including newly synthesized transglycosylation products, in future studies. Although prior research suggests that ScEXG1 may function as a transglycosylase, the specific reaction conditions and product structures have not been fully characterized. Thus, our objectives are to (1) investigate how enzyme and substrate concentrations influence ScEXG1-mediated transglycosylation, (2) assess the transglycosylation efficiency of wild-type ScEXG1, and (3) elucidate the structural characteristics of the transglycosylation product(s) generated by ScEXG1.

## 2 Materials and Methods

### 2.1 Chemicals

Laminaribiose, laminaritriose, and other glucose-derived oligosaccharides standards were purchased from Biosynth Carbosynth (Compton, UK), while trichloroacetic acid, sulfuric acid, and *p*NP-β-d-Glc were obtained from Sigma-Aldrich (St. Louis, MO, USA).

### 2.2 Characterization of ScEXG1

#### 2.2.1 Protein Concentration Assay

Protein concentration was determined using the Bradford method with bovine serum albumin as the standard. In a 96-well plate, 200 μL of Bradford reagent was mixed with 5 μL of the protein sample, and the absorbance was measured at 595 nm.

#### 2.2.2 Purification of Extracellular Native ScEXG1 for Hydrolytic Activity Assay

Extracellular ScEXG1 protein was purified from the culture medium of wild-type *S. cerevisiae* by the KTA protein purification system. The elution profile was monitored at 280 nm, and fractions were collected sequentially. Hydrolytic activity was measured by monitoring the release of *p*-nitrophenol (*p*NP) from *p*NP-β-d-glucopyranoside. A 120 μL reaction mixture containing 5 μL of 10 mM substrate, 5 μL of enzyme, and 110 μL of 50 mM sodium acetate buffer (pH 5.5) was incubated at 40 °C for 10 min. The reaction was terminated with 120 μL of 0.5 M Na_2_CO_3_, and the absorbance was read at 405 nm. One unit (U) of enzyme activity was defined as the amount releasing 1 μmol of *p*NP per minute under these conditions.

#### 2.2.3 Purification of Extracellular Recombinant-6xHis ScEXG1 for Transglycosylation Assays

Transglycosylation assays were performed using His-tagged ScEXG1 produced by recombinant yeast strain BY4741 *exg1*Δ::*pGPD-ScEXG1–6 × His-tCYC1 las21*Δ to enhance secretion and facilitate downstream purification. For recombinant expression, an overnight culture of the ScEXG1–6 × His strain was inoculated into 100 mL of YPD medium (1% yeast extract, 2% peptone, 2% glucose) at an initial OD_6__0__0_ of 0.1 and incubated at 30 °C and 150 rpm for 24 h. Cells were harvested (4500 *g* for 10 min), and the supernatant was concentrated using Amicon centrifugal filters (10 kDa cutoff) at 5000 *g*, 4 °C for 15 min, followed by 0.22 μm filtration. The concentrated supernatant was purified using Ni^2^^+^-NTA affinity chromatography. Elution was carried out using 50–250 mM imidazole in Tris-HCl buffer (20 mM Tris-HCl, 100 mM NaCl, pH 8.0), and purity was confirmed via 10% SDS-PAGE and silver staining. Transglycosylation activity was measured at 30 °C using laminaribiose (4.38–32.85 mM) and purified ScEXG1–6 × His **(**0.02–1 U) in 50 mM sodium acetate buffer (pH 5.5). Reactions were terminated by adding an equal volume of 10% (v/v) trichloroacetic acid. Products were analyzed via thin-layer chromatography (TLC) and HPLC-ESI-MS/MS.

#### 2.2.4 pH and Temperature Profiling

To evaluate pH effects, ScExg1 enzymatic activity was assayed at 40 °C for 30 min using 2.5 mg/mL *p*NPG in citrate-phosphate buffers from pH 2.2 to 8.0. For pH stability, the enzyme was preincubated at 4 °C for 24 h in the same buffers before activity testing. Temperature profiling was done by incubating at different temperatures for 30 min in pH 5.0 buffer, and thermal stability was assessed after 1 h preincubation at each temperature prior to the assay.

### 2.3 TLC

The transglycosylation reactions (20 μL) were analyzed using TLC Silica gel 60 plates (Merck, Darmstadt, Germany). These plates were developed using a solvent mixture consisting of ethyl acetate/acetic acid/water (2:2:1 v/v/v) and were visualized by heating at 125 °C for 10 min after applying a spray of 10% (v/v) sulfuric acid in ethanol.

### 2.4 HPLC-ESI-MS/MS Analysis of ScEXG1 Transglycosylation Products

The transglycosylation products were purified using Supelclean ENVI-Carb SPE cartridges (bed weight 250 mg/3 mL; Sigma-Aldrich, St. Louis, MO, USA). Lactitol was used as an internal standard and added to the transglycosylation products before the HPLC-MS/MS analysis with a final concentration of 1 μg/mL. The analysis was conducted by using an UltiMate 3000 UHPLC system coupled to a Q Exactive hybrid quadrupole-orbitrap mass spectrometer (Thermo Fisher Scientific, Waltham, MA, USA). The transglycosylation products were characterized via ESI-MS/MS in the negative ion mode. The conditions, composition of mobile phases, and ESI source parameters were previously documented in detail.^16^ For the analysis, 10 μL of samples were injected and separated on a Hypercarb with a guard column. A binary gradient of mobile phase A (0.1% NH4OH in water) and B (0.1% NH4OH in 90% acetonitrile) was employed as follows: 0–10 min, 2.5% B to 5% B; 10–20 min, 5% B to 9% B; 20–28 min, 9% B to 30% B; 28–37 min, 30% B to 50% B; 37–40 min, holding at 50% B for 3 min. Subsequently, re-equilibration was performed for 20 min using an isocratic method with 2.5% B. The column temperature was maintained at 40 °C.

Adducts [M – H]^−^ of oligosaccharides were monitored and fragmented using targeted-selected ion monitoring and parallel reaction monitoring modes with the collision set to higher energy C-trap dissociation (HCD) and normalized collision energy ranging from 10 to 30%. Data acquisition and further processing were managed using Xcalibur software (version 4.0; Thermo Fisher Scientific). Mass accuracy was set to 5 ppm for MS^1^ and 10 ppm for MS^2^. Fragment assignments were made following the nomenclature of Domon and Costello.^17^ Linkage determination relied on the fragmentation pattern as established in our experiments and supported by the literature.^18–21^ All cross-ring fragments described in this study are annotated as A-type ions based on the Domon–Costello nomenclature.^17^ In this system, superscript numbers (e.g., 0, 2, 1, 3) indicate which bonds within the sugar ring are cleaved, while the subscript (e.g., A_2_, A_3_) refers to the position of the sugar residue from the nonreducing end. These A-type ions provide structural insights into linkage positions and sugar ring fragmentation patterns.

The analysis of trisaccharide standards was independently confirmed using an HPLC system coupled with a heated electrospray ionization probe and a linear ion trap mass spectrometer (LTQ XL, Thermo Fisher Scientific, Waltham, MA USA) operating in the positive ion mode. Liquid chromatography separation utilized a Hypercarb column (100 × 2.1 mm; 3 μm) at 25 °C with a multistep gradient.^21^ Aqueous solvent A [0.1% (v/v%) aqueous formic acid with 1 × 10^–4^ M NaCl] and organic solvent B (HPLC-grade acetonitrile) were used in the elution gradient: 0–1 min, 0% B; 1–21 min, 10% B; 21–21.1 min, 0% B. The mobile phase flow rate was 300 μL/min. Standards were prepared in ultrapure water.

### 2.5 Statistical Analysis

Data for “Hydrolysis and transglycosylation under different substrate concentrations and time intervals” are presented as mean ± standard deviation from three independent experiments. Statistical significance was determined using one-way analysis of variance (ANOVA), followed by Duncan’s multiple range test for post hoc comparisons. Within the same reaction time condition, means followed by different lowercase letters indicate statistically significant differences (*p* < 0.05).

## 3 Results and Discussion

### 3.1 Characterization of Exg1 Enzyme

Native ScEXG1 was successfully purified from the culture supernatant of *S. cerevisiae*, and its glucosidase activity was confirmed via *p*NP-β-d-glucopyranoside hydrolysis. A single major protein band (∼51.2 kDa) corresponding to ScEXG1 was observed by SDS-PAGE, coinciding with peak enzymatic activity. Similarly, recombinant ScEXG1–6 × His was produced using a secretion-enhanced yeast strain, purified via Ni-NTA affinity chromatography and verified by SDS-PAGE and the activity assay. These procedures enabled subsequent hydrolytic and transglycosylation studies under the defined conditions. Details of purification, SDS-PAGE, and activity profiles are provided in Supplementary Figures S1 and S2.

### 3.2 pH/Temperature Profiles of ScEXG1 Enzyme

The ScEXG1 enzyme has demonstrated the ability to hydrolyze various β(1→3)-glucans and flavonoid glucosides,^22^ as well as β(1→6) glycosidic linkages found in mogrosides.^10^ ScEXG1 purified from wild-type yeast exhibited hydrolytic activity toward *p*NPG, with optimal activity observed at pH 5 (Figure 1A). It also retained over 90% of its activity after 24 h at 4 °C in buffers at pH 4 and pH 5, indicating good pH stability under storage conditions (Figure 1A). The enzyme showed maximum catalytic activity at 50 °C and maintained measurable thermostability after 1 h of incubation at 30–50 °C (Figure 1B).

**Figure 1:**
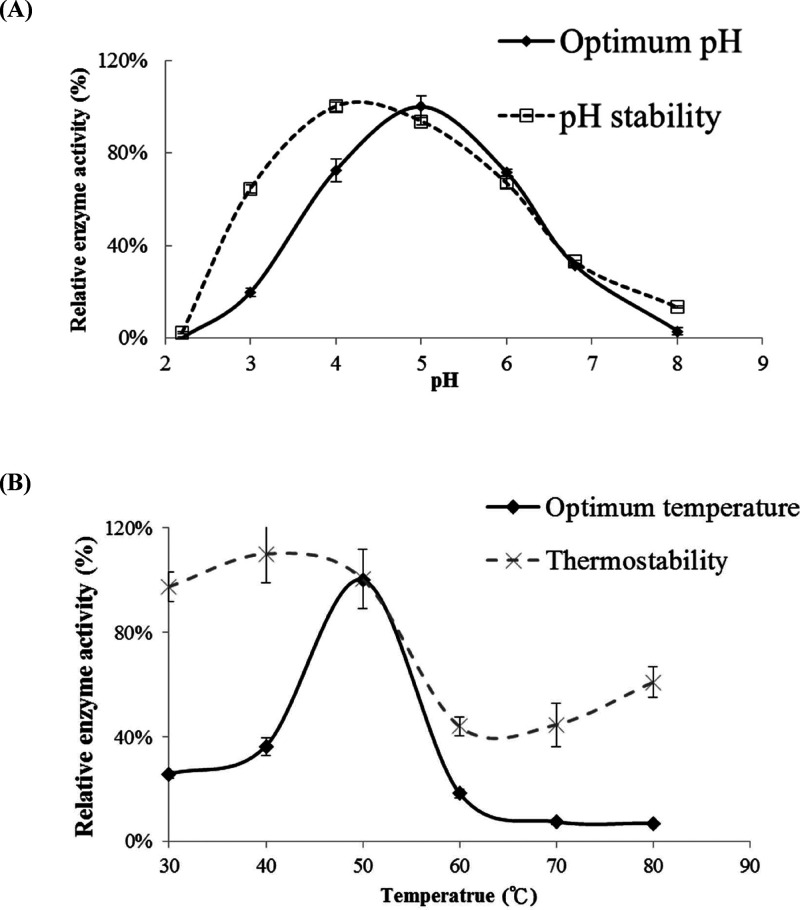
Effects of pH and temperature on the activity and stability of extracellular ScExg1. Enzyme activity was measured using 2.5 mg/mL *p*NPG at 40 °C for 30 min in citrate-phosphate buffer (pH 2.2–8.0). (A) For pH stability, ScExg1 was preincubated at 4 °C for 24 h prior to assay. (B) Temperature-dependent activity was assessed at pH 5.0 for 30 min, and thermal stability was evaluated after preincubation at various temperatures for 1 h.

### 3.3 Hydrolytic and Transglycosylation Activity of ScEXG1–6xHis

The specific activity of ScEXG1–6 × His was determined to be 0.875 ± 0.004 U/mg when assayed with *p*NP-β-d-glucopyranoside (*p*NP-β-d-Glc) at 40 °C and pH 5.0. One unit of enzyme activity (U) is defined as the amount of enzyme that releases 1 μmol of *p*NP per minute under these conditions. While this value appears lower than the specific activities reported for other GH5 enzymes expressed in *E. coli* ^8^ and *Pichia pastoris*,^23^ direct comparisons are limited due to differences in assay parameters. Specifically, our substrate concentration was low, as required for consistent transglycosylation conditions. It is not directly comparable to values measured under saturating conditions. Moreover, additional factors may contribute to the observed differences, including enzyme expression systems, potential differences in folding efficiency, post-translational modifications, presence of a His-tag, and suboptimal hydrolytic conditions.

Nevertheless, these milder conditions are expected to enhance protein stability, which is particularly advantageous for transglycosylation assays that require extended incubation periods. In addition, low-enzyme concentration reduces the rate of hydrolytic cleavage, allowing the intermediate glycosyl-enzyme complex more time to encounter alternative acceptors. A high substrate concentration enhances the likelihood that an acceptor sugar molecule, rather than water, will interact with the glycosyl-enzyme intermediate, thereby promoting glycosidic bond formation over hydrolytic cleavage.^11,24^

To refine the reaction conditions for transglycosylation by ScEXG1–6 × His, we previously applied the response surface methodology (RSM) to screen the influence of substrate concentration and reaction time. The RSM suggested that increased concentrations of laminaribiose (donor) promoted the formation of higher-degree oligosaccharides under mild enzyme activity. To further evaluate this observation, a time-course TLC analysis was conducted using three concentrations of laminaribiose, 4.38 mM, 21.9 mM, and 32.85 mM, all incubated with 0.02 U of ScEXG1–6 × His at 30 °C (Figure 2A–C). The mixture of glucose and laminaribiose displayed clear separation when subjected to the mobile phase consisting of ethyl acetate/acetic acid/water (2:2:1 v/v/v) on the TLC plate. Transglycosylation products, including trisaccharides-DP3 (DP3), were clearly detected across all concentrations but with distinct intensities and profiles. At 4.38 mM (Figure 2A), the reaction yielded modest levels of DP3, with product accumulation stabilizing after 120 min. At 21.9 mM (Figure 2B), a significantly stronger DP3 band was observed, supporting the RSM trend of enhanced transglycosylation with an elevated donor concentration. However, the detailed linkage of these DP3 needs to be further determined. At the highest tested concentration, 32.85 mM (Figure 2C), the DP3 signal remained strong but began to plateau, indicating a potential saturation point or product inhibition at very high substrate concentrations. In our study, faint bands corresponding to tetrasaccharides were observed in Figures 2B and 3C, particularly at later reaction time points. However, due to the low abundance of these products under our experimental conditions, accurate quantification via TLC was not feasible. Therefore, we relied on HPLC-ESI-MS/MS for sensitive and precise structural identification of the tetrasaccharide species. These results support the conclusion that the substrate concentration plays a critical role in shifting ScEXG1–6 × His activity from hydrolysis toward transglycosylation. However, when the enzyme concentration was increased from 0.02 U to 0.2 U, rapid hydrolysis was observed in the early stages of the reaction (0.5 and 1 h), accompanied by a concurrent increase in glucose production (Figure 2D). These findings indicate that both transglycosylation and hydrolysis of laminaribiose by ScEXG1–6xHis occurred simultaneously and that substrate and enzyme concentrations are key factors influencing the balance between these two reactions. Furthermore, the hydrolysis of the transglycosylation products implies that the newly synthesized products may consist of either β (1→3)- and/or β (1→6)-glycosidic linkages as ScEXG1–6xHis possesses the capability to hydrolyze these linkages.^8–10^ However, comprehensive structural characterization of ScEXG1–6xHis transglycosylation products requires confirmation through HPLC-ESI-MS/MS analysis.

**Figure 2:**
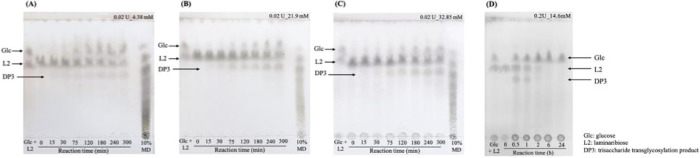
TLC analysis of the profile of the transglycosylation reactions under different ScEXG1–6xHis enzyme or laminaribiose concentrations. (A) 0.02U, 4.38 mM laminaribiose, (B) 0.02U, 21.9 mM laminaribiose, (C) 0.02U, 32.85 mM laminaribiose, and (D) 0.2 U, 14.6 mM. The reactions were carried out at 30 °C in 50 mM sodium acetate buffer (pH 5.5). The reactions were stopped at appropriate time intervals by mixing with an equal volume of 10% (v/v) trichloroacetic acid, and 2–6 μL of the mixture was applied to TLC. The plates were developed with ethyl acetate/acetic acid/water (2:2:1 v/v/v). Lane 1, glucose and laminaribiose standard; (A–C) lanes 2–8; lane 9, 10% (w/v) maltodextrin and (D) lanes 2–7 different reaction time points.

**Figure 3:**
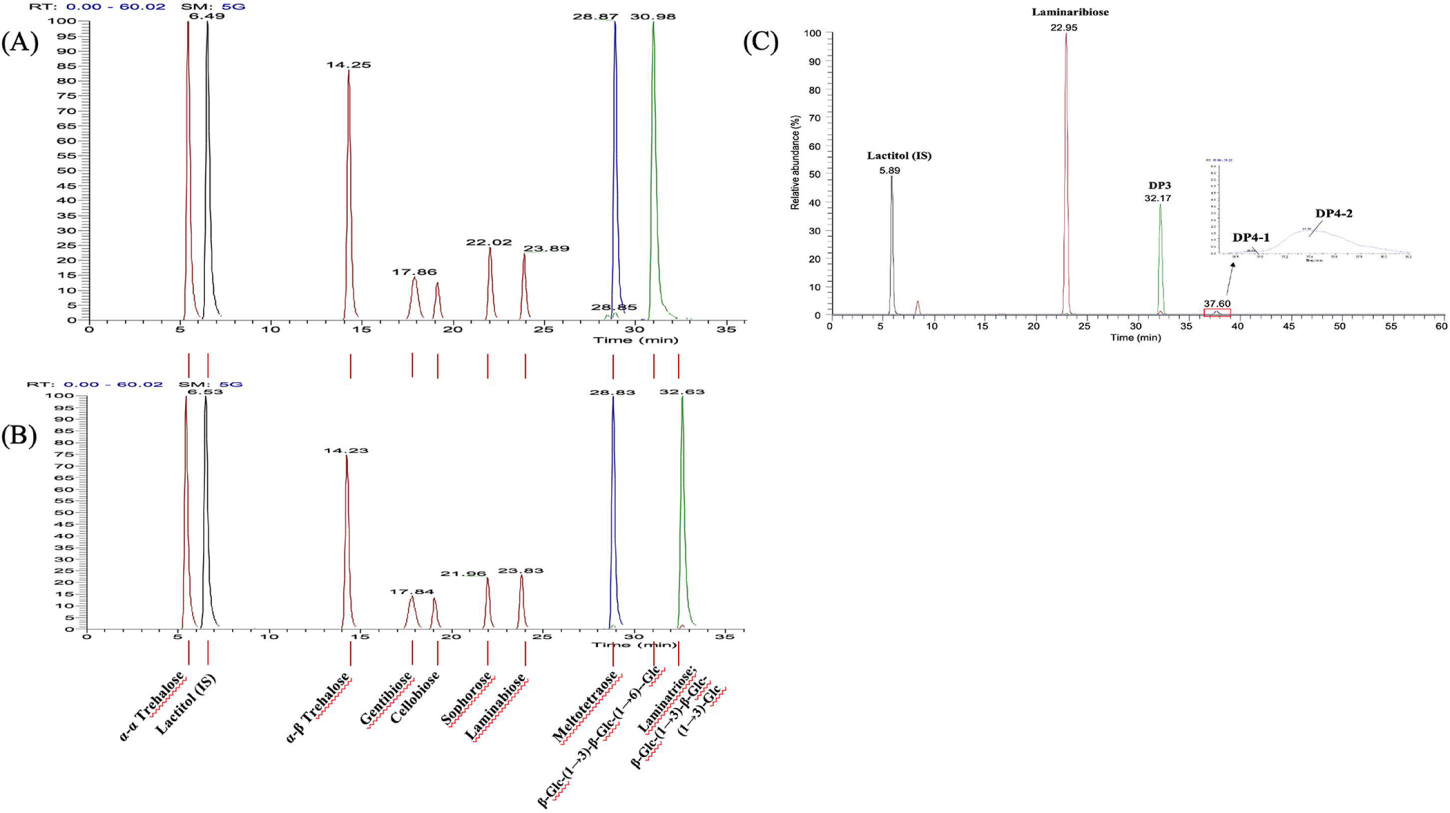
Chromatographic separation of glucose-derived oligosaccharides and transglycosylation products of ScEXG1. (A) Nine glucose-derived oligosaccharides, including the internal standard (IS) lactitol and β-Glc-(1→3)-β-Glc-(1→6)-Glc. (B) Nine glucose-derived oligosaccharides, including the IS lactitol and β-Glc-(1→3)-β-Glc-(1→3)-Glc. (C) Transglycosylation products, including laminaribiose, DP3, DP4–1, and DP4–2 were analyzed along with IS control.

### 3.4 Structure Characterization of ScEXG1–6xHis Transglycosylation Products by HPLC-ESI-MS/MS

To analyze the transglycosylation products, we initiated our study by profiling the structural characteristics of several commercial glucose-derived oligosaccharides, including α-α trehalose, α-β trehalose, sophorose, laminaribiose, cellobiose, and gentibiose, as well as known trisaccharide standards, such as β-Glc-(1→3)-β-Glc-(1→6)-Glc (Figure 3A), laminaritriose known as β-Glc-(1→3)-β-Glc-(1→3)-Glc (Figure 3B), and maltotetraose. The application of HPLC-ESI-MS/MS facilitated the effective separation and analysis of these glucose-derived standard compounds (Figure 3A,B)

Remarkably, ScEXG1 exhibited the ability to perform transglycosylation, resulting in the novel synthesis of DP3 and tetrasaccharides (DP4–1 and DP4–2), respectively (Figure 3C). Our newly identified DP3 does not correspond to any available commercial standards. In addition, DP4–1 and DP4–2 were not readily observed in the initial TLC results, potentially attributable to the method’s lower sensitivity. To gain a more comprehensive understanding of the detailed structures of these newly synthesized oligosaccharides, we required information regarding their molecular ions (*m*/*z* values) and fragmentation patterns. Cross-ring cleavage occurs when the sugar ring itself fragments rather than the glycosidic bond that connects individual sugar units. This type of fragmentation generates A-type ions, which are particularly useful for determining the structure and connectivity of glycosidic linkages. Additionally, cross-ring cleavage events propagating from the reducing end to the nonreducing end provide valuable insights into fragmentation patterns that span across multiple glucose residues, aiding in the comprehensive structural characterization of oligosaccharides.^19–21^ In our result, laminaritriose [β-Glc-(1→3)-β-Glc-(1→3)-Glc, *m*/*z* = 503] was identified, along with low-abundance fragment ions ^1,4^A_2_ (*m*/*z* = 251) and ^0,2^A_2_ (*m*/*z* = 281), which are characteristic of β-1→3 glycosidic linkages (Table 1, Figure 4A). The structural characterization of laminaritriose was confirmed using tandem mass spectrometry (MS/MS), where key diagnostic fragment ions were identified. The presence of the ^1,4^A_2_ fragment (*m*/*z* = 251) resulted from cross-ring cleavage within the second glucose unit, specifically breaking the bond between C1 and C4. Additionally, the detection of ^0,2^A_2_ (*m*/*z* = 281) further supports the β-1,3 linkage arrangement in laminaritriose. This fragment is generated by breaking the bond between the ring oxygen and C2 within the second glucose ring, which is characteristic of β-configured glucans. The combined observation of ^1,4^A_2_ and ^0,2^A_2_ fragments confirms that the glucose units in laminaritriose are exclusively linked by β-1,3 glycosidic bonds. These results collectively provide definitive structural evidence for the β-1,3-linked configuration of laminaritriose.

**Figure 4:**
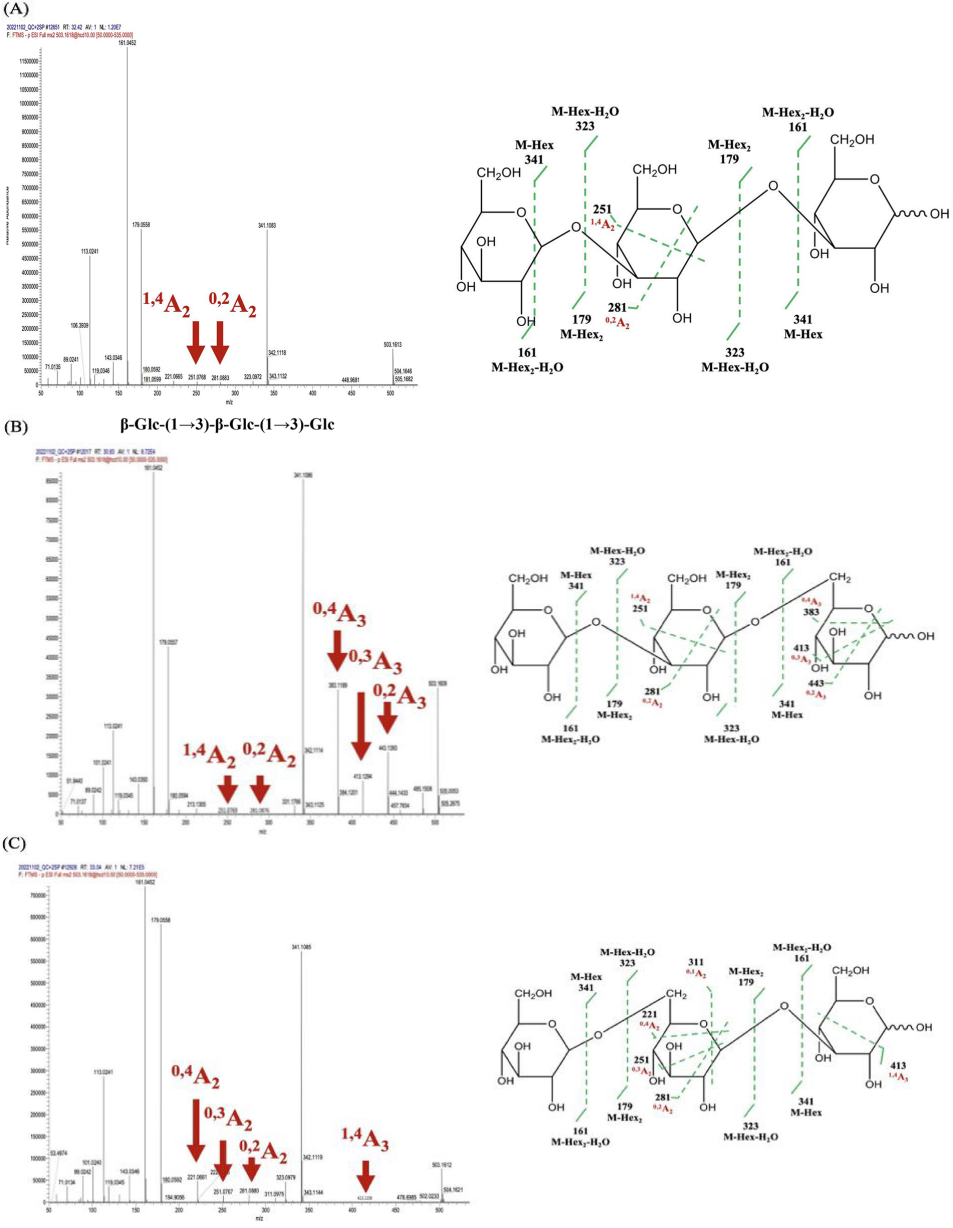
Mass spectrum of the transglycosylation products of ScEXG1. (A) Standards with β-Glc-(1→3)-β-Glc-(1→3)-Glc. (B) Standards with β-Glc-(1→3)-β-Glc-(1→6)-Glc. (C) Trangylcosylation products of ScEXG1, β-Glc-(1→6)-β-Glc-(1→3)-Glc.

**Table 1:** Structural Characterization of the Transglycosylation Products of ScEXG1

no	t_R_ (min)	optimized NCE^a^ (%)	qualitative ion pair^b^ (Nomenclature^c^)	product ion relative abundance (%)	structure identification	reference
gentiobiose	15.9	10	1. 341.1088 → 221.0661 (^0,4^A_2_)	47.6 ± 0.8	β-Glc-(1→6)-Glc	Lin et al.^16^
			2. 341.1088 → 251.0769 (^0,3^A_2_)	0.3 ± 0.1		
			3. 341.1088 → 281.0876 (^0,2^A_2_)	0.7 ± 0.1		
			4. 341.1088 → 311.0978 (^0,1^A_2_)	0.9 ± 0.1		
laminaribiose	22.3	10	1. 341.1088 → 221.0661 (^2,4^A2)	0.4 ± 0.1	β-Glc-(1→3)-Glc	Lin et al.^16^
			2. 341.1088 → 233.0654 (^1,4^A2-H2O)	0.2 ± 0.02		
			3. 341.1088 → 251.0769 (^1,4^A2)	0.1 ± 0.01		
laminaritriose	30.4	10	1. 503.1616 → 251.0769 (^1,4^A_2_)	0.7 ± 0.3		this study
	–30.98		2. 503.1616 → 281.0876 (^0,2^A_2_)	0.4 ± 0.2	β-Glc-(1→3)-Glc-(1→6)-Glc	
			3. 503.1616 → 383.1194 (^0,4^A_3_)	34.6 ± 3.0		
			4. 503.1616 → 413.1338 (^0,3^A_3_)	11.5 ± 2.7		
			5. 503.1616 → 443.1379 (^0,2^A_3_)	23.1 ± 3.7		
laminaritriose	32.05	10	1. 503.1616 → 251.0769 (^1,4^A_2_)	0.3 ± 0.01	β-Glc-(1→3)-Glc-(1→3)-Glc	this study
	–32.63		2. 503.1616 → 281.0876 (^0,2^A_2_)	0.4 ± 0.02		
DP3	32.2	10	1. 503.1616 → 221.0661 (^0,4^A_2_)	8.2 ± 0.5	β-Glc-(1→6)-β-Glc-(1→3)-Glc	this study
			2. 503.1616 → 251.0769 (^0,3^A_2_)	1.9 ± 0.3		
			3. 503.1616 → 281.0876 (^0,2^A_2_)	3.6 ± 0.3		
			4. 503.1616 → 311.0978 (^0,1^A_2_)	1.3 ± 0.6		
			5. 503.1616 → 413.1338 (^1,4^A_3_)	0.1 ± 0.1		
DP4–1	37.1	15	1. 665.2146 → 383.1157 (^0,4^A_3_)	31.9 ± 2.3	β-Glc-(1→3)-β-Glc-(1→6)-β-Glc-(1→3)-Glc	this study
			2. 665.2146 → 443.1379 (^0,2^A_3_)	27.6 ± 8.9		
DP4–2	37.6	15	1. 665.2146 → 221.0666 (^0,4^A_2_)	3.9 ± 2.0	β-Glc-(1→6)-β-Glc-(1→3)-β-Glc-(1→3)-Glc	this study
			2. 665.2146 → 251.0773 (^0,3^A_2_)	0.7 ± 0.6		
			3. 665.2146 → 281.0877 (^0,2^A_2_)	0.5 ± 0.6		
			4. 665.2146 → 311.0982 (^0,1^A_2_)	0.1 ± 0.1		

a Collision mode: HCD, precursor ion [M – H]^−^.

b Detected *m*/*z*.

c According to Domon and Costello.^17^

To further investigate the influence of glycosidic linkage patterns on fragmentation behavior, a second trisaccharide standard, β-Glc-(1→3)-β-Glc-(1→6)-Glc (*m*/*z* = 503), revealed prominent fragmentation ions, including ^0,4^A_3_ (*m*/*z* 383), ^0,3^A_3_ (*m*/*z* 413), and ^0,2^A_3_ (*m*/*z* 443) (Figure 4B and Table 1), complementing the earlier detection of minor fragmentation ions ^1,4^A_2_ and ^0,2^A_2_. The presence of the ^0,4^A_3_ fragment (*m*/*z* = 383), which corresponds to cross-ring cleavage between the ring oxygen and C4 of the third glucose unit, suggests that this residue exhibits increased ring flexibility. This observation is consistent with a β-1,6 linkage, which allows greater rotational freedom compared to β-1,3 or β-1,4 linkages. The ^0,3^A_3_ ion (*m*/*z* = 413), resulting from the cleavage between the ring oxygen and C3, further supports this assignment as β-1,6-linked glucose residues typically produce stronger ^0,3^A and ^0,4^A fragment ions. Additionally, the detection of the ^0,2^A_3_ ion (*m*/*z* = 443), formed by cleavage between the ring oxygen and C2, confirms that the third glucose unit is structurally influenced by a β-1,6 glycosidic bond. These results highlight the distinct fragmentation behavior of β-1,6-linked glucose residues compared to fully β-1,3-linked oligosaccharides. The presence of prominent ^0,4^A_3_ and ^0,3^A_3_ ions, along with the characteristic ^0,2^A_3_ ion, serves as a key indicator of β-1,6 glycosidic linkages, distinguishing this trisaccharide from other β-glucan-derived oligosaccharides.

To further elucidate the structure of the newly synthesized DP3, a detailed analysis was performed. The first glycosidic linkage of DP3, starting from the nonreducing end was determined as β-1→6 based on the presence of specific cross-ring cleavage ions. The presence of ^0,4^A_2_ (*m*/*z* = 221), ^0,3^A_2_ (*m*/*z* = 251), ^0,2^A_2_ (*m*/*z* = 281), and ^0,1^A_2_ (*m*/*z* = 311) indicates that the second glucose unit in DP3 underwent characteristic cross-ring cleavage events, confirming a β-1→6 linkage (Figure 4C and Table 1). Additionally, the detection of ^1,4^A_3_ (*m*/*z* = 413), a cross-ring cleavage fragment derived from the third glucose unit, provides further confirmation of the β-1,3 linkage at this position (Figure 4C and Table 1). Unlike the first linkage, no cross-ring cleavage was observed for the second glycosidic linkage, suggesting that this region of the molecule was more resistant to ring cleavage. This absence of A-type ions indicates that the second linkage is likely β-1→3, which is further supported by the overall fragmentation pattern (Figure 4C). The absence of detection on glycosidic bond cleavage ions (B- and C-type ions) further confirmed the order of glucose residues in DP3 as β-Glc-(1→6)-β-Glc-(1→3)-β-Glc. As for transglycosylation specificity, Nakatani et al. demonstrated a shift in specificity from β-1,3 to β-1,6 by a GH5 glycosynthase variant.^24^ In contrast, our study focuses on wild-type ScEXG1, and we observed the formation of β-Glc-(1→6)-β-Glc-(1→3)-β-Glc oligosaccharides, as confirmed by HPLC-ESI-MS/MS analysis. This reflects the natural promiscuity of the enzyme rather than the engineered specificity.

In our analysis, we also identified other minor transglycosylated products, including DP4–1 and DP4–2. DP4–1 exhibited no noticeable cross-ring fragmentation on the first and third linkages from the nonreducing end, thus confirming them as 1→3 linkages. The second linkage from the nonreducing end was determined as 1→6 based on the presence of cross-ring cleavage ions ^0,4^A_3_ (*m*/*z* 383) and ^0,2^A_3_ (*m*/*z* 443), leading to the characterization of DP4–1 as β -Glc-(1→3)-β-Glc-(1→6)-β-Glc-(1→3)-β-Glc (Figure 5A). Interestingly, DP4–2 featured the first linkage from the nonreducing end determined as 1→6 based on the presence of cross-ring cleavage ions ^0,4^A_2_ (*m*/*z* 221), ^0,3^A_2_ (*m*/*z* 251), ^0,2^A_2_ (*m*/*z* 281), and ^0,1^A_2_ (*m*/*z* 311) (Figure 5B). The second and third linkages from the nonreducing end were identified as 1→3 based without the detection of cross-ring cleavage ions. Consequently, DP4–2 was assigned the structure β -Glc-(1→6)-β-Glc-(1→3)-β-Glc-(1→3)-β -Glc (Figure 5B). While an earlier study suggested the potential transglycosylation of laminaribiose into laminaritriose by ScEXG1, no structural confirmation of this transglycosylation trisaccharide product was provided.^8^ In our study, we initially demonstrated that ScEXG1 can indeed transglycosylate larminaribiose into DP3 and various DP4 oligosaccharides. However, our findings raise intriguing questions as laminaritriose was not detected as a product of ScEXG1 in this work. We speculated that this absence of laminaritriose detection may be attributed to the higher affinity of ScEXG1 for hydrolyzing β(1→3)-linkages compared to β(1→6) linkages as previously reported.^8^ Consequently, laminaritriose might have been hydrolyzed at a faster rate, preventing its detection as a sole product. Nevertheless, the mechanisms behind the synthesis of terminal β(1→6) linkages and the formation of minor product DP4–1 remain unclear at this stage. The hydrolysis and transglycosylation activities of ScEXG1 are also dependent on the enzyme concentration and the availability of laminaribiose as the substrate. Our findings indicate that when lower enzyme concentrations (0.02 U) were employed in the reaction, a 2 h reaction period led to the accumulation and detection of three distinct transglycosylation products (DP3, DP4–1, and DP4–2) (Figures 4C and 5). Conversely, with higher enzyme concentrations (0.2 and 1 U), laminaribiose showed a rapid reduction within 15 min, indicative of laminaribiose hydrolysis (data not shown). This outcome underscores that an increased enzyme presence in the reaction offers more active sites for catalytic hydrolysis, resulting in a hydrolysis rate surpassing that of transglycosylation product synthesis and consequently causing a reduction in transglycosylation products.^11^ In contrast, at lower enzyme concentrations, the hydrolysis of laminaribiose and the transglycosylation products exhibited reduced activity, leading to the gradual accumulation of transglycosylation products and an apparent increase in the transglycosylation efficiency. It is worth noting that transglycosylation is a kinetically controlled reaction. Increasing the concentration of the acceptor molecule reduces the effective water activity in the reaction system, which can shift the catalytic balance of ScExg1 away from hydrolysis and toward transglycosylation.^11^

**Figure 5:**
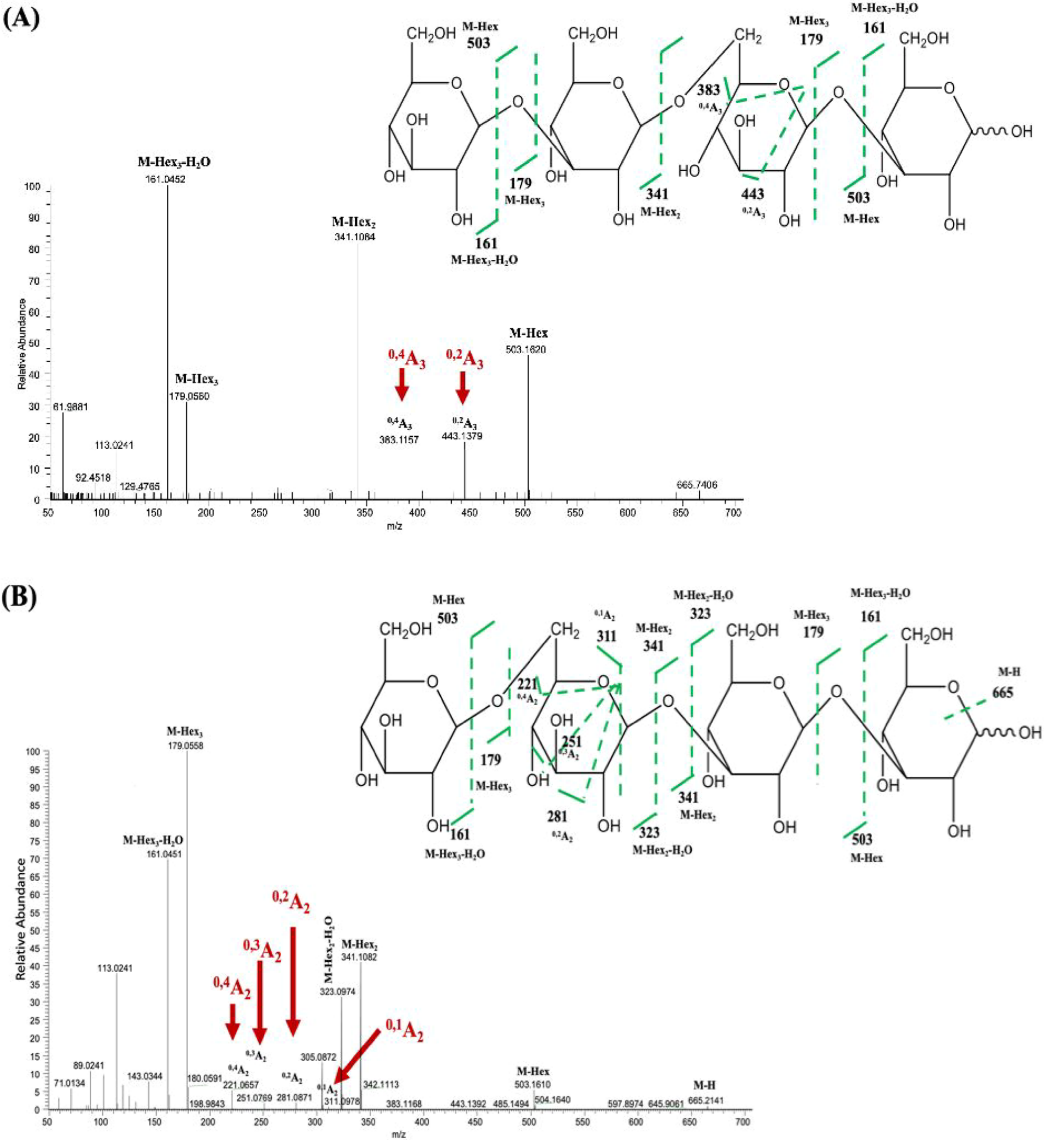
Mass spectrum of the transglycosylation products of ScEXG1. (A) DP4–1 and (B) DP4–2. Selected ion [M – H]^−^ = 665.2146, collision energy NCE 15%. All analysis were set with parameters as follows: isolation window ± 4 *m*/*z*, collision-induced dissociation HCD, resolution 17,500.

### 3.5 Effect of Substrate Concentration and Reaction Time on Hydrolysis and Transglycosylation

We further investigated the influence of varing laminaribiose concentrations (4.38, 21.9, and 32.85 mM) under low concentrations of ScEXG1 (0.02 U) on the transglycosylation efficiency. Our results demonstrated that transglycosylation products could be formed under all tested substrate conditions, with DP3 consistently being the predominant product. At 21.9 mM laminaribiose, DP3 reached a peak concentration of 270.70 ± 10.32 nmol/mL after 180 min, which resulted in 1.24% of transglycosylation. Minor amounts of DP4–1 and DP4–2 were also observed but could not be quantified due to their low abundance. Therefore, only DP3 concentration was reported in Table 2 as the representative transglycosylation product. In contrast, higher laminaribiose concentrations (21.9 and 32.85 mM) consistently resulted in lower hydrolysis efficiency across all time points (75, 120, and 180 min), with no significant differences observed between these two concentrations. The highest hydrolysis level observed was 123.39 ± 42.58 nmol/mL of glucose at 180 min, equivalent to a hydrolysis yield of 1.41 ± 0.49% (Table 2). Despite the overall low hydrolysis, transglycosylation was favored at higher substrate concentrations. Notably, TLC analysis confirmed that transglycosylation products were still detectable up to 5 h, even under low-enzyme conditions (Figure 2A–C). The relatively modest yield is likely due to the relatively water-rich and enzyme-limited conditions selected to facilitate structural elucidation over maximal productivity. Overall, these results highlight that ScEXG1 can promote both hydrolytic and synthetic reactions with the balance between them influenced by the substrate concentration and the reaction time.

**Table 2:** Hydrolysis and Transglycosylation under Different Substrate Concentrations and Time Intervals^a^ ^,^ ^b^

reaction time (min)	laminaribiose (mM)	glucose (nmol/mL)	DP3 (nmol/mL)	hydrolysis (%)^c^	transglycosylation(%)^d^
75	4.38	98.06 ± 16.81^a^	24.26 ± 0.36^a^	1.11 ± 16.83^b^	0.56 ± 0.01^b^
	21.9	136.06 ± 17.62^a^	151.26 ± 2.65^c^	0.31 ± 0.04^a^	0.69 ± 0.01^c^
	32.85	123.39 ± 49.79^a^	120.21 ± 3.38^b^	0.18 ± 0.06^a^	0.36 ± 0.01^a^
120	4.38	113.16 ± 19.17^a^	28.65 ± 2.17^a^	1.29 ± 0.22^b^	0.65 ± 0.05^a^
	21.9	166.26 ± 3.68^b^	222.54 ± 9.84 ^b^	0.38 ± 0.01^a^	1.01 ± 0.04^b^
	32.85	216.92 ± 22.69^b^	216.26 ± 26.60^b^	0.25 ± 0.03^a^	0.49 ± 0.06^a^
180	4.38	123.39 ± 42.58^a^	30.79 ± 1.97^a^	1.41 ± 0.49^b^	0.71 ± 0.05^a^
	21.9	216.92 ± 18.66^b^	270.70 ± 10.32^b^	0.5 ± 0.04^a^	1.24 ± 0.05^b^
	32.85	238.36 ± 27.04^b^	261.22 ± 17.97^b^	0.27 ± 0.03^a^	0.54 ± 0.04^a^

a The data are shown as mean ± standard deviation from three independent experiments and analyzed by one-way ANOVA followed by Duncan’s multiple range test.

b ^a–c^Mean followed by different lowercase letters represent significant differences for the values in the same reaction time conditions (*p* < 0.05).

c Hydrolysis (%) was calculated as $\left(\frac{\mathrm{Glu}\cos e\;\left(\frac{n\mathrm{mol}}{\mathrm{mL}}\right)}{\mathrm{La}\min\mathrm{aribiose}\;\left(\frac{n\mathrm{mol}}{\mathrm{mL}}\right)}\right)÷2\times 100\%$ . The ratio between laminaribiose:glucose is considered as 1:2.

d Transglycosylation (%) was obtained from $\left(\frac{\mathrm{DP}3\;\left(\frac{n\mathrm{mol}}{\mathrm{mL}}\right)}{\mathrm{La}\min\mathrm{aribiose}\;\left(\frac{n\mathrm{mol}}{\mathrm{mL}}\right)}\right)\times 100\%$ .

In the present study, we not only demonstrated the transglycosylation activity of ScEXG1 using laminaribiose as the substrate but also, for the first time, elucidated the structure of novel oligosaccharides synthesized by β-Glc-(1→6)-β-Glc-(1→3)-Glc (DP3), β-Glc-(1→3)-β-Glc-(1→6)-β-Glc-(1→3)-Glc (DP4–1), and β-Glc-(1→6)-β-Glc-(1→3)-β-Glc-(1→3)-Glc (DP4–2). Structural assignments were supported by HPLC-ESI-MS/MS analysis using PGC chromatography, high-resolution mass spectrometry, and linkage-informative cross-ring fragmentation profiling. Co-elution and fragmentation comparisons with commercial standards (e.g., laminaribiose, laminaritriose, and maltotetraose) further strengthened the structural determination. While NMR remains the gold standard for definitive linkage confirmation, our data robustly support the presence of isomeric DP3 and DP4 structures, highlighting the structural diversity achievable through ScEXG1-mediated transglycosylation. Although the biological activities and potential applications of these newly identified oligosaccharides are yet to be determined, our study establishes a foundation for future exploration.

Moreover, to leverage ScEXG1 for large-scale oligosaccharide production, strategies such as site-directed mutagenesis or directed evolution could be considered. These approaches aim to abolish the hydrolytic activity while enhancing the transglycosylation activity of ScEXG1, enabling efficient linkage-specific synthesis of these oligosaccharides. The findings from our study hold promise for the future development of innovative oligosaccharides with potential applications as prebiotics.

## Supplementary Material



## Notes

Purification of extracellular native ScEXG1 for hydrolytic activity assay and purification of extracellular recombinant-6xHis ScEXG1 for transglycosylation assays (PDF)
