# Supplementary material for: Structural Analysis of Laminarin-Derived Oligosaccharides Produced by Transglycosylation of Exo-β-1,3-glucanase ScEXG1 from Saccharomyces cerevisiae
Source: J Agric Food Chem. 2025 Dec 24;74(1):1053–62. doi: 10.1021/acs.jafc.5c04471 (PMC12814337; doi:10.1021/acs.jafc.5c04471)
Supplement: Supplementary file 1 [file jf5c04471_si_001.pdf]

“Supporting Information”

Structural analysis of laminarin-derived oligosaccharides produced by transglycosylation of the exo-β-1,3-glucanase ScEXG1 from *Saccharomyces cerevisiae*

Szu-Yu Kuo[a #] , Chih-Chieh Lin[a #] , Hsin-Jo Chang[a], Reuben Wang[bc], Pei-Yin Lin[d], Ting-Jang Lu[a,d*], Yi-Chen Lo[a*]

[a] Institute of Food Science and Technology, National Taiwan University, No. 1, Section 4, Roosevelt Rd., Taipei, Taiwan. TW 106319

[b] Institute of Food Safety and Health, College of Public Health, National Taiwan University, Taipei, Taiwan. TW 100025

[c] Master of Public Health Program (MPH), College of Public Health, National Taiwan University, Taipei City, Taiwan. TW 100025

[d] College of Bioresources and Agriculture, Joint Center for Instruments and Researches, No. 81, Changxing St., Da-an Dist., Taipei, Taiwan. TW 106038

*** Correspondence:** Yi-Chen Lo, Ph. D.; Ting-Jang Lu, Ph. D.

Address: Institute of Food Science and Technology, National Taiwan University, No. 1, Section 4, Roosevelt Rd., Taipei 10617, Taiwan.

E-mail: loyichen@ntu.edu.tw

Phone: +886-2-33664123

E-mail: tjlu@ntu.edu.tw

Phone: +886-2-33664131

[#] **Equal contribution: Szu-Yu Kuo; Chih-Chieh Lin**

Supplementary Data

S1. Purification of Extracellular Native ScEXG1 for Hydrolytic Activity Assay.

Extracellular ScEXG1 protein purified from the culture medium of wild-type *S. cerevisiae* by ÄKTA Protein Purification System. The elution profile was monitored at 280 nm, and fractions were collected sequentially. Hydrolytic activity was measured by monitoring the release of p-nitrophenol (pNP) from pNP-β-D-glucopyranoside. A 120 μL reaction mixture containing 5 μL of 10 mM substrate, 5 μL enzyme, and 110 μL of 50 mM sodium acetate buffer (pH 5.5) was incubated at 40°C for 10 min. The reaction was terminated with 120 μL of 0.5 M Na_2CO_3, and absorbance was read at 405 nm. One unit (U) of enzyme activity was defined as the amount releasing 1 μmol of pNP per minute under these conditions.

Results:

ScExg1 protein was purified from *S. cerevisiae* using the ÄKTA Protein Purification System. Protein-containing fractions were collected and analyzed for both protein content and enzymatic activity. As shown in Figure S1A, multiple protein fractions were eluted during purification. These were then resolved by SDS-PAGE to evaluate molecular weight distribution (Figure S1B). A distinct protein band was observed at ~51.2 kDa in fraction 42, consistent with the theoretical molecular weight of ScExg1 predicted from the Saccharomyces Genome Database (SGD). To confirm enzymatic identity, a pNPG-based glucosidase activity assay was performed on each eluted fraction. The highest activity was found in fraction 42 (Figure S1C)., which corresponded with the SDS-PAGE band at ~51.2 kDa, thereby confirming successful purification and functional identity of ScExg1.

(S1A)

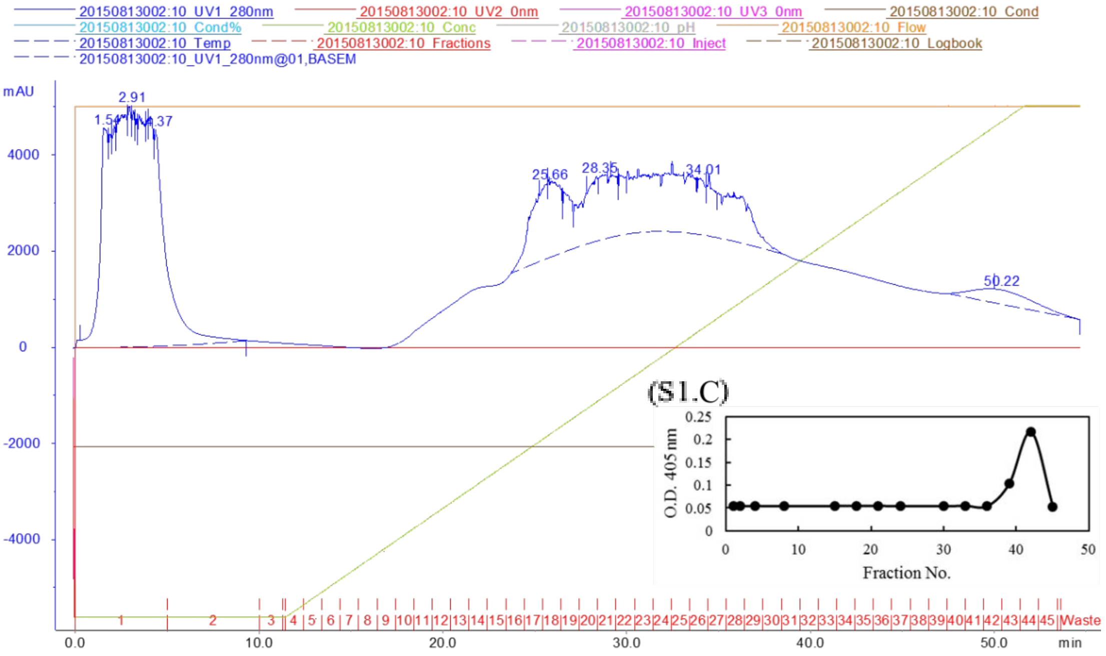

(S1 B)

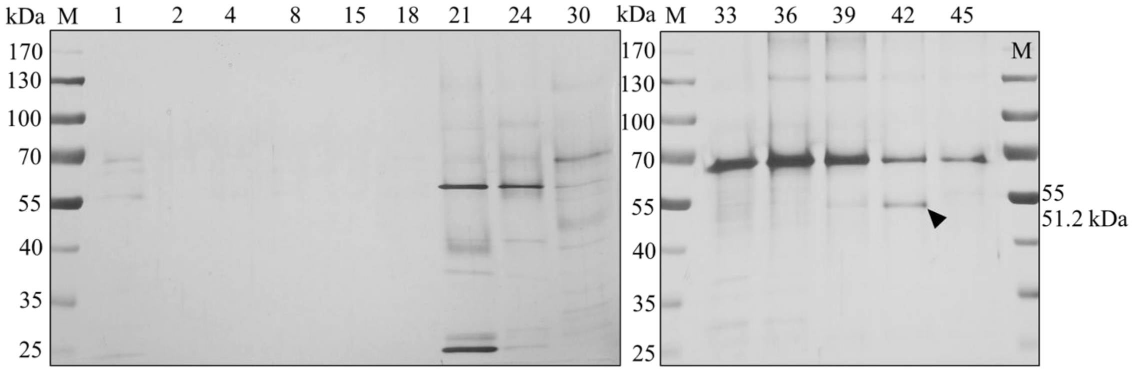

Figure S1. Purification of extracellular native ScEXG1 for hydrolytic activity assay. (S1A) The analysis of protein patterns in fractions 1 to fraction 45 from the ÄKTA Protein Purification System. Numbers represent fraction numbers from ÄKTA Protein Purification Systems. (S1B) Fraction 42 contains possible *Sc*Exg1 with a molecular weight of approximately 51-52 kDa, indicated by the arrowhead, which was identical to the information retrieved from SGD. (S1C) The test of glucosidase activity using *p*NPG in each eluted fraction. Fraction no.42 shows the highest glucosidase activity.

S2 Purification of Extracellular Recombinant-6xHis ScEXG1 for

Transglycosylation Assays.

Transglycosylation assays were performed using His-tagged ScEXG1 produced by recombinant yeast strain BY4741 *exg1Δ*::pGPD-ScEXG1-6×His-tCYC1 *las21Δ* to enhance secretion and facilitate downstream purification. For recombinant expression, an overnight culture of the ScEXG1-6×His strain was inoculated into 100 mL YPD medium (1% yeast extract, 2% peptone, 2% glucose) at an initial OD_{600} of 0.1 and incubated at 30°C, 150 rpm for 24 h. Cells were harvested (4500 × g for 10 min), and the supernatant was concentrated using Amicon® centrifugal filters (10 kDa cut-off) at 5000 × g, 4°C for 15 min, followed by 0.22 μm filtration. The concentrated supernatant was purified using Ni^{2+}-NTA affinity chroma-tography. Elution was carried out using 50-250 mM imidazole in Tris-HCl buffer (20 mM Tris-HCl, 100 mM NaCl, pH 8.0) and purity was confirmed via 10% SDS-PAGE and silver staining. Transglycosylation activity was measured at 30°C using laminaribiose (4.38–32.85 mM) and purified ScEXG1-6×His (0.02-1 U) in 50 mM sodium acetate buffer (pH 5.5). Reactions were terminated by adding an equal volume of 10% (v/v) trichloroacetic acid. Products were analyzed via thin-layer chromatography (TLC) and

HPLC-ESI-MS/MS.

Results:

Saccharomyces cerevisiae BY4741 *exg1Δ* mutant, complemented with the wild-type *EXG1* gene under the control of the GPD promoter and CYC1 terminator, and tagged with a 6×His sequence. This resulted in the strain BY4741 *exg1Δ::pGPD-ScEXG1-6×His-tCYC1*. To enhance the secretion of Exg1 into the culture medium, the *LAS21* gene was further deleted. This genetic modification yielded the strain BY4741 *exg1Δ::pGPD-ScEXG1-6×His-tCYC1 las21Δ*, which is capable of secreting EXG1-6×His into the medium for subsequent collection and purification.

Protein purification was performed with a Ni-NTA affinity column. Briefly, the cells were cultured in a total volume of 100 mL with a starting cell density OD_{600} of 0.1 for 24 h at 30ºC with 150 rpm shaking. The collected culture was concentrated to c.a. 8 mL followed by purification with Ni-NTA affinity column. The collected proteins were analyzed by 10% SDS-PAGE gels, and visualized with silver staining (Figure S2). It was observed that the resin-bound ScEXG1 protein was eluted with 50-250 mM imidazole in Tris-HCl buffer (20 mM Tris-HCl, 100 mM NaCl, pH 8.0), and its molecular mass was also in line with the theoretical value (~52.4 kDa) calculated

from its amino acid composition. To remove the imidazole from the collected proteins (50-250 mM imidazole fractions), they were dialyzed against 50 mM sodium acetate (pH 5.5) several times, and concentrated with a Amicon® centrifugal filter to 0.5 mL aliquot. Protein concentration was determined with the Bradford method, using BSA as the standard. Protein activity was tested against *p*NP-β-Glucose. One unit of enzyme was defined as the production of one μmol glucose per minute at 40ºC.

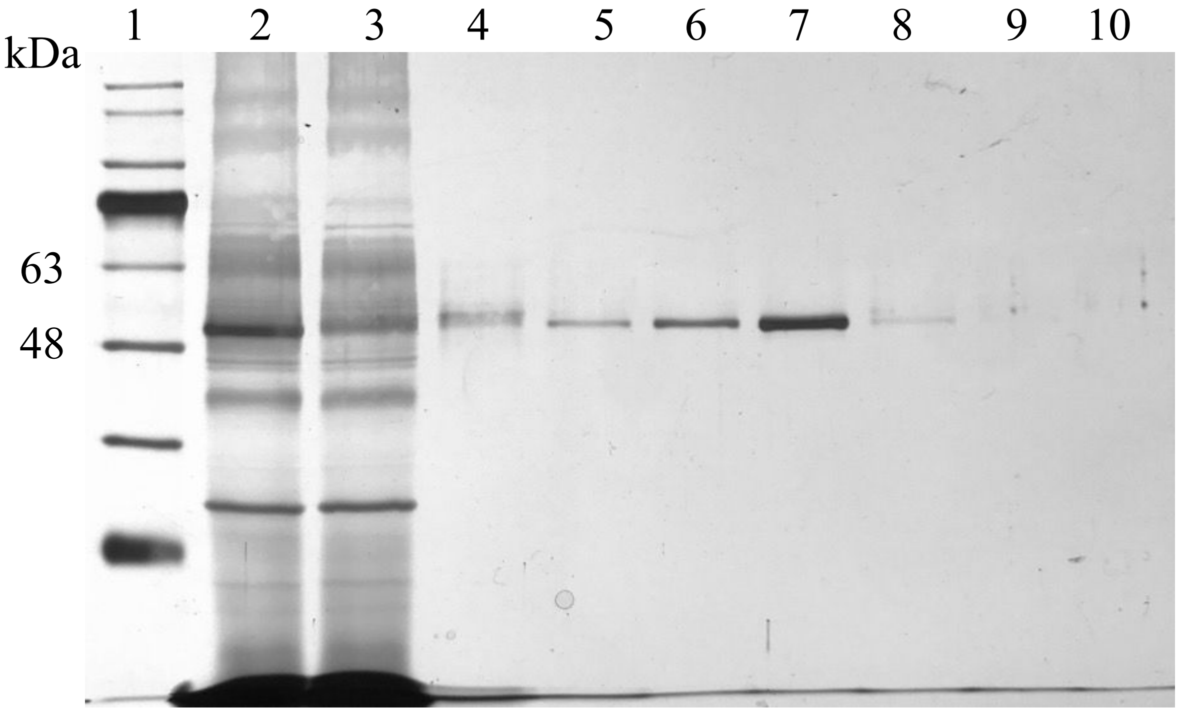

Figure S2. Recombinant ScExg1-6×His protein purification

Recombinant **ScExg1-6×His** protein expected size is 52.4 kDa. The proteins were analyzed with 10% SDS-PAGE gels and visualized with silver staining.

Lane 1, protein marker; lane 2, protein lysate; lane 3, flow through from Ni-NTA column; lane 4, elution with washing buffer; lane 5-10, proteins eluted with 50 mM (lane 5-6), 250 mM (lane 7-8), and 500 mM (lane 9-10) of imidazole in Tris-HCl.
